# Visualizing Mutation-Specific Differences in the Trafficking-Deficient Phenotype of Kv11.1 Proteins Linked to Long QT Syndrome Type 2

**DOI:** 10.3389/fphys.2018.00584

**Published:** 2018-05-23

**Authors:** Allison R. Hall, Corey L. Anderson, Jennifer L. Smith, Tooraj Mirshahi, Claude S. Elayi, Craig T. January, Brian P. Delisle

**Affiliations:** ^1^Department of Physiology, University of Kentucky, Lexington, KY, United States; ^2^Cellular and Molecular Arrhythmia Research Program, University of Wisconsin–Madison, Madison, WI, United States; ^3^Department of Molecular and Functional Genomics, Genomic Medicine Institute, Geisinger Clinic, Danville, PA, United States; ^4^Department of Cardiology, Gill Heart Institute, University of Kentucky, Lexington, KY, United States

**Keywords:** K^+^ channel, trafficking, long QT syndrome, arrhythmia, Kv11.1, hERG

## Abstract

*KCNH2* encodes the Kv11.1 α-subunit that underlies the rapidly activating delayed-rectifier K^+^ current in the heart. Loss-of-function *KCNH2* mutations cause long QT syndrome type 2 (LQT2), and most LQT2-linked missense mutations inhibit the trafficking of Kv11.1 channel protein to the cell surface membrane. Several trafficking-deficient LQT2 mutations (e.g., G601S) generate Kv11.1 proteins that are sequestered in a microtubule-dependent quality control (QC) compartment in the transitional endoplasmic reticulum (ER). We tested the hypothesis that the QC mechanisms that regulate LQT2-linked Kv11.1 protein trafficking are mutation-specific. Confocal imaging analyses of HEK293 cells stably expressing the trafficking-deficient LQT2 mutation F805C showed that, unlike G601S-Kv11.1 protein, F805C-Kv11.1 protein was concentrated in several transitional ER subcompartments. The microtubule depolymerizing drug nocodazole differentially affected G601S- and F805C-Kv11.1 protein immunostaining. Nocodazole caused G601S-Kv11.1 protein to distribute into peripheral reticular structures, and it increased the diffuse immunostaining of F805C-Kv11.1 protein around the transitional ER subcompartments. Proteasome inhibition also affected the immunostaining of G601S- and F805C-Kv11.1 protein differently. Incubating cells in MG132 minimally impacted G601S-Kv11.1 immunostaining, but it dramatically increased the diffuse immunostaining of F805C-Kv11.1 protein in the transitional ER. Similar results were seen after incubating cells in the proteasome inhibitor lactacystin. Differences in the cellular distribution of G601S-Kv11.1 and F805C-Kv11.1 protein persisted in transfected human inducible pluripotent stem cell derived cardiomyocytes. These are the first data to visually demonstrate mutation-specific differences in the trafficking-deficient LQT2 phenotype, and this study has identified a novel way to categorize trafficking-deficient LQT2 mutations based on differences in intracellular retention.

## Introduction

Long QT syndrome (LQTS) is a disorder characterized by delayed ventricular repolarization, prolongation of the QT interval on an electrocardiogram (ECG), and an increased risk for the ventricular arrhythmia Torsades de Pointes (Crotti et al., 2008). LQT2 is a major form of LQTS, which is caused by loss-of-function (LOF) mutations in the *KCNH2* gene (a.k.a the *human ether-a-go-go related gene* or *hERG*) (Curran et al., 1995; Sanguinetti et al., 1996; Smith et al., 2016). *KCNH2* encodes the pore-forming Kv11.1 α-subunits that conduct the rapidly activating delayed-rectifier K^+^ current (*I*_Kr_) in the heart (Trudeau et al., 1995).

The majority of LQT2-linked mutations are missense, and heterologous expression studies demonstrate that ∼90% of LQT2 missense mutations decrease the intracellular transport (trafficking) of Kv11.1 channels to the cell surface membrane (Zhou et al., 1998a; Ficker et al., 2000a; Anderson et al., 2006, 2014). Trafficking-deficient LQT2 mutations are postulated to disrupt Kv11.1 α-subunit protein folding/assembly and cellular quality control (QC) mechanisms in endoplasmic reticulum (ER) prevent their trafficking early in the secretory pathway (Zhou et al., 1998a, 1999; Ficker et al., 2003; Delisle et al., 2005; Gong et al., 2005; Walker et al., 2007, 2010; Foo et al., 2016). A key finding is several drugs that bind to Kv11.1 channels and block *I*_Kr_ (e.g., E-4031) can act as “pharmacological chaperones” to improve the trafficking and functional expression for many LQT2 channels (pharmacological correction) (Zhou et al., 1999; Ficker et al., 2002; Anderson et al., 2006, 2014). This is an important finding because it indicates that some trafficking-deficient LQT2 channels are promising targets for therapeutic intervention to improve delivery of channels to the surface membrane. Data suggest that pharmacological chaperones bind to the high affinity drug-binding site in the Kv11.1 pore to stabilize Kv11.1 channel conformations suitable for ER export into the secretory pathway (Zhou et al., 1999; Ficker et al., 2002; Ellgaard and Helenius, 2003; Delisle et al., 2005; Gong et al., 2006).

We previously studied the cellular QC mechanisms that prevent the trafficking of the LQT2-linked missense mutations Gly601Ser (G601S) early in the secretory pathway. In control conditions, most of the G601S-Kv11.1 protein is excluded from ER exit sites (ERES) and sequestered in a microtubule-sensitive ER compartment that contains the transitional ER protein BAP31 (Furutani et al., 1999; Ficker et al., 2002; Wakana et al., 2008; Smith et al., 2011, 2013). This transitional ER compartment did not contain other ER or ER Golgi Intermediate proteins, including calnexin, proteins with the ER retention KDEL sequence, the ER-associated degradation (ERAD) protein derlin-1, the ERES protein Sec31, or the ER-Golgi intermediate compartment 53 kDa protein (ERGIC-53). Importantly, we found that incubating cells in the pharmacological chaperone E-4031 directly increased the trafficking of the sequestered G601S-Kv11.1 channels to the cell surface membrane (Smith et al., 2013). In this study we investigate the cellular QC control mechanisms that prevent the trafficking of the LQT2-linked missense mutation F805C (Ficker et al., 2002). Unlike G601S (which is located on the extracellular side of the Kv11.1 α-subunit in the pore domain), F805C is located on the intracellular portion of the Kv11.1 α-subunit in the cyclic nucleotide-binding domain (CNBD) and does not undergo pharmacological correction with E-4031 because it likely impairs Kv11.1 tetramer assembly (Ficker et al., 2002; Delisle et al., 2003). We tested the hypothesis that the ER QC control mechanisms that prevent the trafficking of G601S- and F805C-Kv11.1 proteins are different.

## Materials and Methods

### Cell Lines and Drug Exposure

**FIGURE 1:**
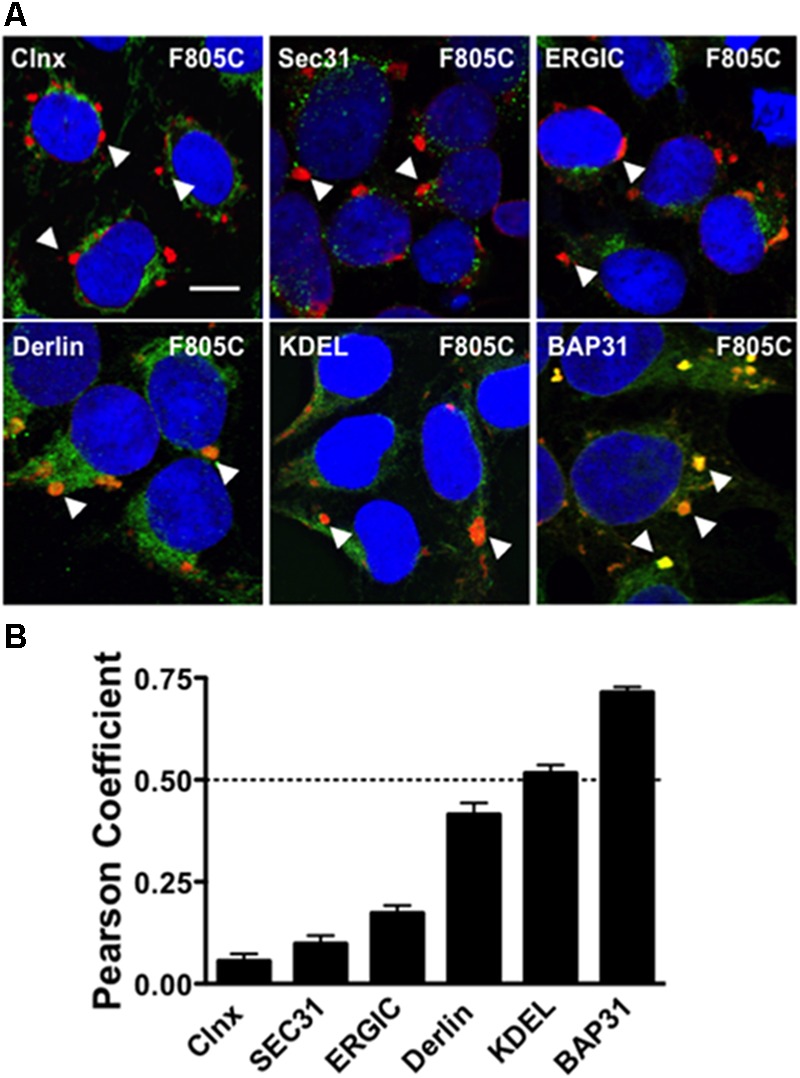
F805C selectively co-localizes with BAP31. **(A)** Representative confocal images of HEK293 cells expressing F805C-Kv11.1 protein showing the overlap between anti-Kv11.1 and anti-ER/ERGIC protein immunostaining. Anti-Kv11.1 immunostaining is shown in red; anti-calnexin (*n* = 12 images), anti-Sec31 (*n* = 19 images), anti-ERGIC-53 (*n* = 19 images), anti-derlin-1 (*n* = 18 images), anti-KDEL (*n* = 15 images), or anti-BAP31 (*n* = 17 images) is shown as green; overlapping immunostaining of red and green signals of similar intensities is shown as yellow; and the cell nuclei are labeled blue. The arrowheads highlight the distinct immunostaining pattern of F805C-Kv11.1 protein, and the scale bar represents 10 μm. **(B)** The mean Costes’ Pearson Coefficient (PC) for the image data sets are shown. PC values at or below the dashed line are (PC = 0.5) indicative of weak or no co-localization. Two independent cultures were tested for each condition.

**FIGURE 2:**
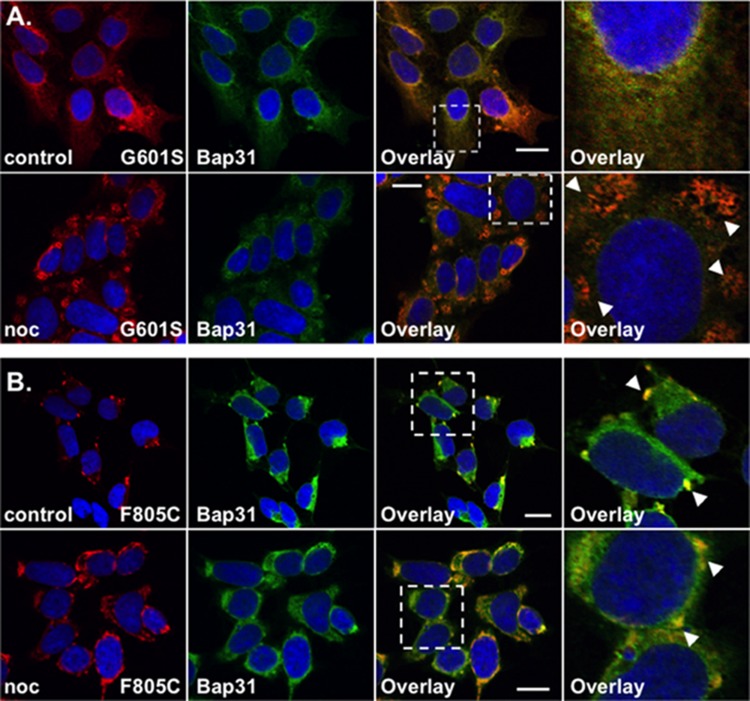
G601S- and F805C-Kv11.1 proteins show distinct immunostaining patterns. Shown are representative confocal images of HEK293 cells expressing **(A)** G601S- or **(B)** F805C-Kv11.1 protein immunostained with anti-Kv11.1 (red, first column), anti-Bap31 (green, second column) in control conditions (top row; *n* = 13 images for G601S and *n* = 17 images for F805C) or after incubation in nocodazole (bottom row; *n* = 15 images for G601S and *n* = 16 images for F805C). The overlay images are also shown (overlapping immunostaining is yellow, third column), and the white dashed box portion of the overlay image is shown in larger detail (fourth column). The nuclei are labeled blue. The arrowheads in **(A)** highlight the reticular immunostaining pattern of G601S-Kv11.1 protein after nocodazole treatment, the arrowheads in **(B)** highlight the unique immunostaining pattern of F805C-Kv11.1 protein, and the scale bar represents 10 μm. Two independent cultures were tested for each condition.

**FIGURE 3:**
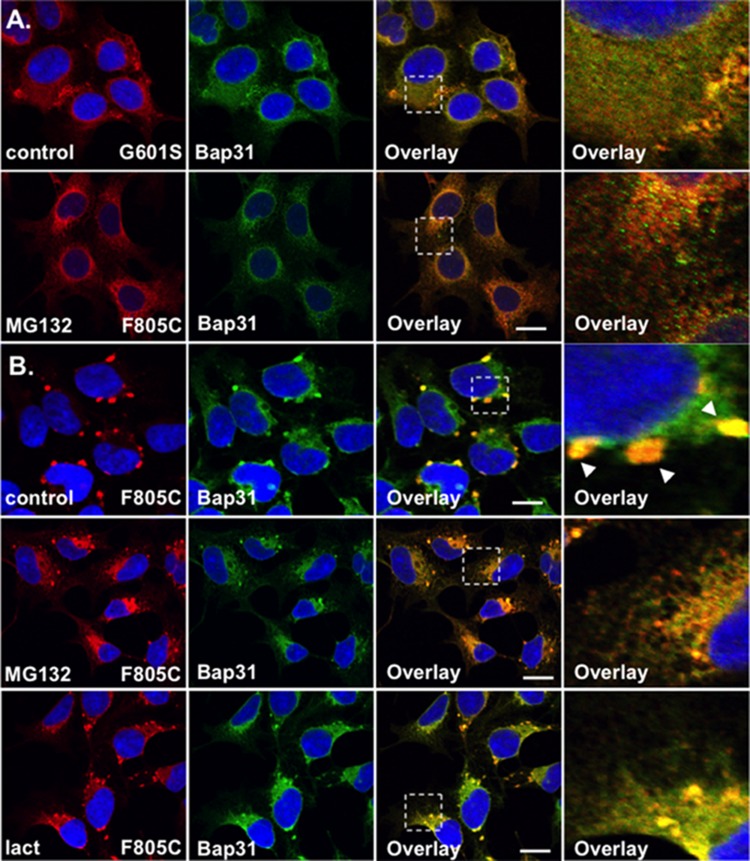
F805C-Kv11.1 protein immunostaining is sensitive to proteasome inhibition. Shown are representative images of HEK293 cells expressing **(A)** G601S- or **(B)** F805C-Kv11.1 protein immunostained with anti-Kv11.1 (red, first column), anti-Bap31 (green, second column), the overlay (co-localization is shown as yellow, third column), and the white dashed box portion of the overlay in more detail (fourth column). The nuclei are shown in blue and the scale bars represent 10 μm. The top row of images in **(A,B)** show control conditions (*n* = 13 images for G601S and *n* = 17 images for F805C) and the second row of images in **(A,B)** show cells after incubation in 5 μM MG132 for 5 h (*n* = 15 images for G601S and *n* = 17 images for F805C). The arrowheads in **(B)** highlight the immunostaining pattern of F805C-Kv11.1 protein in control conditions. The bottom row of images show F805C-Kv11.1 protein immunostaining after incubation in 5 μM lactacystin (*n* = 17 images) for 5 h. Two independent cultures were tested for each condition.

**FIGURE 4:**
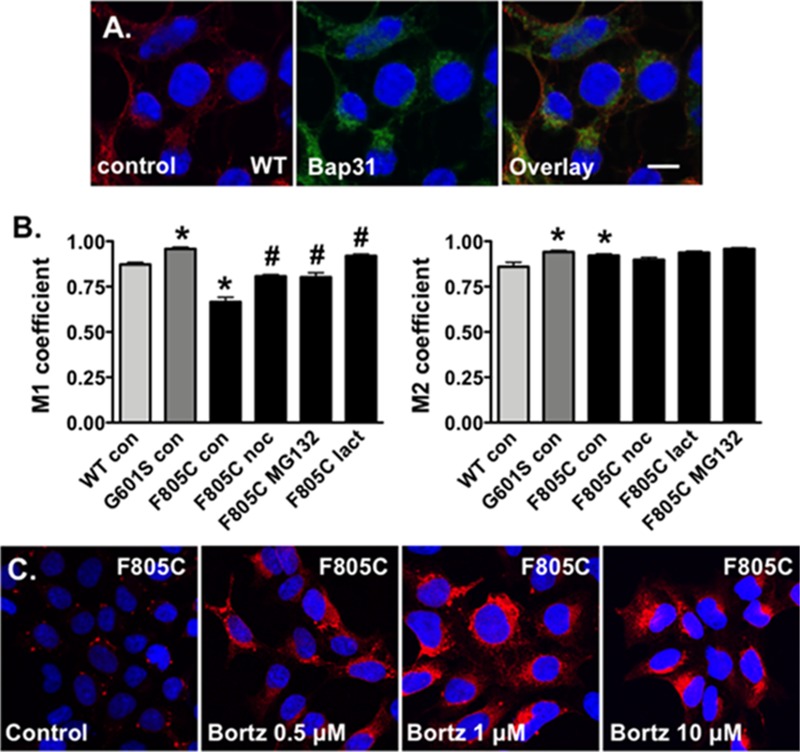
Proteasome inhibition increases the fraction of Kv11.1 signal overlapping with BAP31 signal. **(A)** Shown are representative confocal images of HEK293 cells expressing WT-Kv11.1 protein immunostained with anti-Kv11.1 (red, first column), anti-Bap31 (green, second column) in control conditions. The nuclei are labeled blue, and the scale bar represents 10 μm. **(B)** Shown is the graph for the MCC that describes the fraction of Kv11.1 signal in compartments containing the BAP31 signal (M1, left graph), as well as the fraction of BAP31 signal in compartments containing Kv11.1 signal (M2, right graph). We compared M1 and M2 for cells stably expressing WT-, G601S-, or F805C-Kv11.1 protein in control conditions (*n* = 19, 13, and 17 images, respectively, ^∗^*p* < 0.05 compared to cells expressing WT-Kv11.1 in control conditions). We also compared the M1 or M2 in cells expressing F805C-Kv11.1 in control conditions to cells incubated in nocodazole (F805C noc, *n* = 16 images), MG132 (F805C MG132, *n* = 17 images), or lactacystin (F805C lact, *n* = 17 images). At least two independent cultures were tested for each condition (^#^*p* < 0.05) compared to cells expressing F805C-Kv11.1 protein in control conditions. **(C)** Shown are representative images of cells expressing F805C-Kv11.1 protein immunostained with anti-Kv11.1 (red) in control conditions or in different concentrations of bortezomib for 5 h. The nuclei are labeled blue. The effect of bortezomib on F805C-Kv11.1 immunostaining was confirmed in two independent test cultures (*n* = 5–11 images per condition).

**FIGURE 5:**
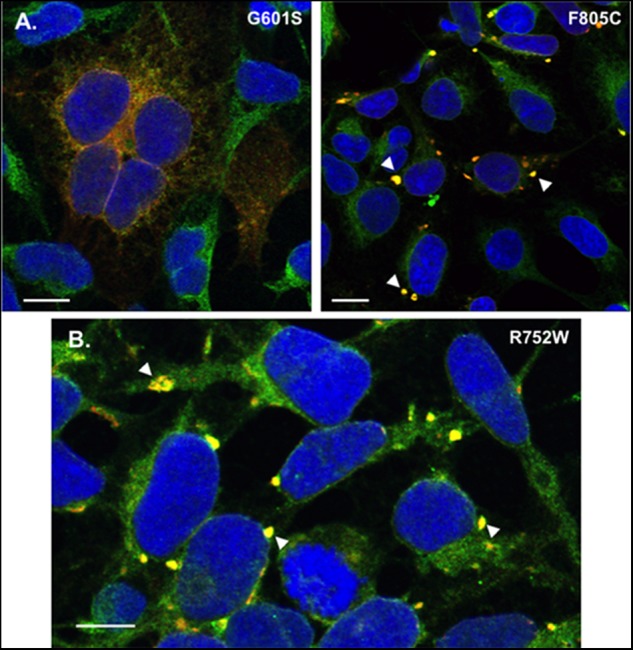
Transient transfection of G601S- or F805C-Kv11.1 does not alter mutation-specific differences in immunostaining pattern, and cells stably expressing R752W-Kv11.1 protein show similar immunostaining patterns as cells expressing F805C-Kv11.1 protein. **(A)** Representative confocal images of HEK293 cells transiently expressing G601S-Kv11.1 (left) or F805C-Kv11.1 (right) protein showing the overlapped anti-Kv11.1 (red) and anti-BAP31 immunostaining (green) (*n* = 12 images for each, from 2 independent cultures). Overlapping immunostaining of red and green signal of similar intensities is shown as yellow and the cell nuclei are labeled blue. The transfection efficiency is <100%, so not all cells were expressing Kv11.1 protein. **(B)** Representative confocal images of HEK293 cells stably expressing R752W-Kv11.1 protein showing the overlap between anti-Kv11.1 (red) and anti-BAP31 immunostaining (green). Overlapping immunostaining of red and green signal of similar intensities is shown as yellow and the cell nuclei are labeled blue. The arrowheads highlight the immunostaining pattern of R752W-Kv11.1 protein, and the scale bar represents 10 μm. The mean Costes’ Pearson Coefficient (PC) for the image data is 0.65 ± 0.01 (*n* = 11 images from 2 independent cultures).

The Human Embryonic Kidney 293 (HEK293) cells used in this study stably express Kv11.1 channel proteins (wild type or WT, G601S, R752W, or F805C) and they have been previously described (Zhou et al., 1998b; Delisle et al., 2003; Anderson et al., 2006). Cells are cultured at 37°C (5% CO_2_) in MEM supplemented with 10% Fetal Bovine Serum (FBS, Invitrogen, Carlsbad, CA, United States) and geneticin. The data shown in **Figures 1–4**, **5B** are obtained from stably expressing cell lines. For some experiments we transiently transfected G601S- or F805C-Kv11.1 plasmid cDNA in HEK293 cells. Transfections were performed similar to that previously described (Anson et al., 2004), 3 μg of Kv11.1 plasmid cDNA was transfected with the Superfect Transfection Reagent (Qiagen, Germantown, MD, United States). The data for **Figure 5A** are from transiently expressing HEK293 cells. We also used transiently transfected iCell Cardiomyocytes (Cellular Dynamics International, Madison, WI, United States). iCell Cardiomyocytes were transiently transfected with green fluorescent protein (GFP) tagged Kv11.1 cDNA plasmids (3 μg) using the Viafect (Promega, Madison, WI, United States) transfection reagent and the protocol for iCell Cardiomyocytes. The GFP cDNA is located in-frame and upstream of the Kv11.1 protein start site. For pharmacological studies, we incubated cells in nocodazole (20 μM) (Millipore, Sigma, St. Louis, MO, United States), bortezomib, lactacystin, and MG132 (Millipore, Sigma, St. Louis, MO, United States), or E-4031 (10 μM) (Alomone Laboratories, Jerusalem, Israel). Nocodazole, bortezomib, lactacystin or MG132 were dissolved in DMSO and added to the MEM (final DMSO concentration was <0.1%). E-4031 was dissolved in water. Control studies were done in parallel with equivalent amounts of vehicle (DMSO or water) only.

### Immunocytochemistry and Confocal Imaging

Immunocytochemistry and confocal imaging experiments were done similar to those previously described (Smith et al., 2011, 2013). HEK293 cells or iCell Cardiomyocytes were plated in 35 mm tissue culture plates containing collagen-coated coverslips. Cells were fixed with 4% buffered paraformaldehyde for 10 min, permeabilized with triton X-100 (0.1%) for 10 min, and rinsed in 0.75% glycine buffer for 10 min to quench background fluorescence. The cells were incubated with 2 mL blocking solution (10% goat serum in PBS) for 1 h to block non-specific binding sites, and the cells were subsequently incubated with the appropriate primary antibodies: anti-Kv11.1 (1:1000, Alomone Laboratories, Jerusalem, Israel), anti-calnexin (1:1000, Abcam, Cambridge, MA, United States), anti-KDEL (1:1000, Abcam, Cambridge, MA, United States), anti-Sec31a (1:100, BD Labs, Franklin Lakes, NJ, United States), anti-Bap31 (1:500, Abcam, Cambridge, MA, United States), and anti-ERGIC-53 (1:1000, Alexis Biochemicals, Plymouth Meeting, PA, United States) mixed with blocking solution at room temperature. Excess antibody was washed off with three 10 min long rinses of blocking solution. The cells were then incubated for 1 h with the appropriate Highly Cross-absorbed Alexa Fluor antibodies (1:500, Invitrogen Inc., Carlsbad, CA, United States). The cells were washed four times for 10 min with blocking solution alone. After the final wash, the coverslips were mounted onto a slide using ProLong Gold with DAPI mounting medium (Invitrogen, Carlsbad, CA, United States). For iCell Cardiomyocytes experiments, we prepared the cells as described above but utilized the fluorescence signal from GFP for imaging analyses. Imaging was performed at the University of Kentucky Imaging Facility using a Leica TSP SP5 confocal microscope (Leica, Wetzler, Germany). HEK293 cells or iCell Cardiomyocytes did not show any labeling with any of the secondary antibodies when used alone (data not shown). Data are shown as representative single z-scan images for each fluorescence signal (fluorescent images). Overlapping signals of similar intensities colored as red and green are shown as yellow and green and purple are shown as white.

Co-localization analyses were performed using ImageJ^1^. We calculated the Pearson Coefficient (PC) and the Manders Colocalization Coefficients (MCC) for some experiments. The PC is the goodness of fit for the linear regression describing the relation between overlapping signal intensities. We calculated the coefficients using the intensity thresholds defined by the Costes’ approach to minimize the contribution of noise. Using the Costes PC approach, the values range between 0 (no co-localization) to 1 (100% co-localization). Weak co-localization is defined as having PC values < 0.5 (Bolte and Cordelieres, 2006). The MCC calculates the fraction of overlap between the signals even if the intensities of the signals are very different from one another. The MCC also vary from 0 (no overlap) to 1 (complete overlap) and are calculated for both signals (M1 and M2). We use the MCC to describe the fraction of Kv11.1 signal in compartments containing the BAP31 signal (M1), or to describe the fraction of BAP31 signal in compartments containing Kv11.1 signal (M2).

### Statistics

A one-way ANOVA was performed on data sets. In order to identify which experimental groups differed from control, *post hoc* Bonferroni’s tests on selected data sets were performed (Prism, La Jolla, CA, United States). Significance was reported at *p* < 0.05.

## Results

### F805C-Kv11.1 Protein Selectivity Co-localizes With BAP31

We initially tested whether the trafficking-deficient F805C-Kv11.1 protein co-localized with different ER/ERGIC proteins (**Figure 1A**) in HEK293 cells by calculating the Costes PC. Confocal data showed that F805C-Kv11.1 protein immunostaining localized into several discrete compartments throughout the cell. Based on the PC analysis, the F805C-Kv11.1 protein did not (or only weakly) co-localize with the ER lectin chaperone calnexin (Hammond et al., 1994), the ER Golgi Intermediate Complex (ERGIC) protein ERGIC-53 (Schweizer et al., 1993), the ERES protein Sec31 (Salama et al., 1997), the ERAD protein derlin-1 (Lilley and Ploegh, 2004), or ER proteins that contain the KDEL ER retention signal sequence (Munro and Pelham, 1987) (**Figure 1B**). However, F805C-Kv11.1 protein co-localized with the transitional ER protein BAP31. These data suggest that F805C-Kv11.1 protein is concentrated in the transitional ER.

### G601S- and F805C-Kv11.1 Proteins Show Differences in Protein Immunostaining

We previously showed that G601S-Kv11.1 protein is sequestered in microtubule-dependent ER QC compartment with BAP31 (Smith et al., 2011, 2013). We directly compared the anti-Kv11.1 protein immunostaining pattern of HEK293 cells expressing G601S-Kv11.1 or F805C-Kv11.1 protein in control conditions or after treatment in the microtubule depolymerizing agent nocodazole (20 μM) for 2 h. Similar to what was previously reported, cells expressing G601S-Kv11.1 protein showed a peri-nuclear immunostaining pattern that diffused into the cell periphery and co-localized with BAP31 in control conditions (**Figure 2A**; PC = 0.62 ± 0.02 in control *n* = 13 images) (Smith et al., 2011, 2013). Incubating cells in nocodazole caused G601S-Kv11.1 and BAP31 protein to redistribute together into several peripheral reticulated membrane compartments (PC = 0.72 ± 0.02 after nocodazole, *n* = 15 images). In control conditions, the F805C-Kv11.1 protein immunostaining pattern was qualitatively different than the G601S-Kv11.1 protein immunostaining pattern (**Figure 2B**). Similar to **Figure 1A**, the F805C-Kv11.1 protein immunostaining was concentrated in several compartments that co-localized with BAP31 protein (PC = 0.71 ± 0.01 in control *n* = 17 images). Nocodazole treatment did not cause F805C-Kv11.1 protein to redistribute to reticulated membrane compartments, but it modestly increased the amount of diffuse F805C-Kv11.1 protein immunostaining, still co-localizing with BAP31 (PC = 0.77 ± 0.02 after nocodazole, *n* = 16 images). We conclude that unlike G601S-Kv11.1 protein, F805C-Kv11.1 protein likely concentrates to distinct sub-compartments in the transitional ER.

### F805C-Kv11.1 Protein Immunostaining Is Disrupted by Proteasome Inhibition

The WT Kv11.1 α-subunit is synthesized in the ER as a ∼135 kDa N-linked glycoprotein, and once the WT-Kv11.1 α-subunit traffics to the Golgi apparatus, it is terminally glycosylated to a ∼155 kDa glycoprotein (Zhou et al., 1998b; Petrecca et al., 1999). Studies show that only 60% of the 135 kDa WT-Kv11.1 protein is processed to the terminally glycosylated 155 kDa form. The fraction of WT-Kv11.1 protein that fails to mature is degraded via the ERAD (ubiquitin-proteasome) pathway (Gong et al., 2005). In the presence of proteasome inhibitors, there is an increase in the immature form of WT-Kv11.1 protein, and a decrease in the efficiency in the ER dislocation of WT-Kv11.1 protein in ERAD pathway (Gong et al., 2005). Therefore, we tested whether the proteasome inhibitor MG132 impacted G601S- or F805C-Kv11.1 protein immunostaining. We incubated HEK293 cells expressing G601S- or F805C-Kv11.1 protein in the proteasome inhibitor MG132 (5 μM) for 5 h (**Figures 3A,B**). This incubation time is based on previous reports that investigated the impact that proteasome inhibitors had on the ER retention of proteins in other studies (Kamhi-Nesher et al., 2001; Spiliotis et al., 2002; Wakana et al., 2008). Compared to control cells, incubating cells in MG132 did not appear to alter G601S-Kv11.1 protein immunostaining or its co-localization with BAP31 (PC = 0.57 ± 0.02 after MG132, *n* = 15 images). In contrast, treating cells expressing F805C-Kv11.1 protein dramatically transformed the protein immunostaining pattern from several compartments to a peri-nuclear pattern that diffused into the cell periphery (**Figure 3B**). Although incubating cells in MG132 dramatically altered the distribution of F805C-Kv11.1 protein, the F805C-Kv11.1 protein still co-localized with BAP31 (PC = 0.78 ± 0.02 after MG132, *n* = 17 images). We next determined if another proteasome inhibitor similarly affected the immunostaining pattern of cells expressing F805C-Kv11.1 protein. Incubating cells expressing F805C-Kv11.1 protein in the proteasome inhibitor lactacystin for 5 h also altered the F805C-Kv11.1 protein immunostaining pattern similar to cells incubated with MG132 (**Figure 3B**). Moreover, incubating cells in lactacystin did not alter F805C-Kv11.1 protein co-localization with BAP31 (PC = 0.72 ± 0.03 after lactacystin, *n* = 17 images). These data indicate that F805C-Kv11.1 protein concentrates in sub-compartments of the transitional ER that are sensitive to proteasome inhibition.

### Nocodazole, Lactacystin, and MG132 Increase the Fractional Overlap of F805C-Kv11.1 Protein With BAP31 and Immunostaining

The Costes’ PC quantifies the co-localization between Kv11.1 and BAP31 protein immunostaining by measuring the goodness of fit for the relation between overlapping signal intensities. An additional approach for confocal image analysis is to calculate the MCC, which provides a measure of how much one signal overlaps with a second signal. Qualitatively, the major effect of incubating cells expressing F805C-Kv11.1 protein in nocodazole (**Figure 2B**), MG132, or lactacystin (**Figure 3B**) was to increase the overlap of Kv11.1 protein with BAP31 immunostaining, therefore we quantified the MCC between Kv11.1 protein and BAP31.

Manders Colocalization Coefficients analyses have not been done for cells expressing Kv11.1 proteins. Therefore we imaged cells stably expressing WT-Kv11.1 protein (**Figure 4A**) and compared the M1 and M2 with cells expressing G601S- or F805C-Kv11.1 protein. The data show the M1 for cells expressing WT-Kv11.1 protein is smaller than that of cells expressing G601S-Kv11.1 protein (**Figure 4B**). In other words, cells expressing WT-Kv11.1 protein show less overlap of Kv11.1 protein with BAP31 compared to cells expressing G601S-Kv11.1 protein. These data are consistent with more G601S-Kv11.1 protein retained in the transitional ER compartment. In contrast, the M1 for cells expressing WT-Kv11.1 protein is greater than F805C-Kv11.1 protein. This is because F805C-Kv11.1 protein concentrates in several small transitional ER subcompartments. We conclude that mutation-specific differences in the trafficking-deficient LQT2 phenotype can be quantified by differences in the fraction of Kv11.1 protein overlapping with BAP31.

In contrast the M2 for cells expressing WT-Kv11.1 protein is smaller than cells expressing G601S- or F805C-Kv11.1 protein (**Figure 4B**). This means that more BAP31 overlapped with Kv11.1 protein in cells expressing G601S- or F805C-Kv11.1 protein. This is because cells expressing WT-Kv11.1 protein appear to have more Kv11.1 protein in the cell surface membrane (**Figure 4A**).

We next determined how incubating cells expressing F805C-Kv11.1 protein in nocodazole or the proteasome inhibitors affected the M1 and M2 (**Figure 4B**). Compared to control cells expressing F805C-Kv11.1, incubating cells expressing F805C-Kv11.1 protein in the proteasome inhibitors increased M1. These data demonstrate that nocodazole and the proteasome inhibition increased the amount of F805C-Kv11.1 protein in the larger transitional ER compartment. We did not see any significant changes in M2, indicating that incubating cells in nocodazole or proteasome inhibitors did affect the high amount of BAP31 overlap with F805C-Kv11.1 protein.

The effect of proteasome inhibitors on the immunostaining patterns of F805C-Kv11.1 protein was striking. Inhibiting the proteasome pathway is an approach in the treatment of certain cancers, including myeloma and mantle cell lymphoma (Chen et al., 2011). Bortezomib is the first FDA approved proteasome inhibitor to be implemented in cancer treatment. Therefore, we also determined the impact that different concentrations of bortezomib had on the immunostaining pattern of F805C-Kv11.1 protein (**Figure 4C**). We found that, similar to MG132 and lactacystin, incubating cells in different concentrations of bortezomib for 5 h transformed the F805C-Kv11.1 protein immunostaining pattern to a diffuse peri-nuclear pattern.

### G601S- and F805C-Kv11.1 Proteins Show Differences in Protein Immunostaining in Transiently Transfected Cells

One possible reason for the immunostaining difference between the cell lines expressing G601S- and F805C-Kv11.1 could be due to differences in protein expression. The unique immunostaining pattern of F805C-Kv11.1 protein might be because the mutant protein levels are too high for a normal functioning of the cellular QC system. Therefore we immunostained HEK293 cells transiently transfected with equal amounts of G601S- or F805C-Kv11.1 plasmid DNA. Importantly, the mutation-specific immunostaining pattern of G601S- and F805C-Kv11.1 proteins were similar to what was seen in stably expressing cells (**Figure 5A**). These data suggest that the mutation-specific difference in immunostaining was not due to differences in mutant Kv11.1 cDNA levels.

We previously showed that cells stably expressing different LQT2-linked mutations in the Kv11.1 pore region (A614V- or N629D-Kv11.1) exhibit a diffuse immunostaining pattern that co-localizes with BAP31 similar to G601S-Kv11.1 protein (Smith et al., 2013). We determined whether R752W-Kv11.1 (another LQT2-linked mutation that is located intracellular CNBD, impairs Kv11.1 tetramer assembly, and does not undergo pharmacological correction with E-4031) exhibited a similar immunostaining pattern as cells expressing F805C-Kv11.1 protein. Imaging cells stably expressing R752W-Kv11.1 protein showed that it also concentrated in transitional ER subcompartments (**Figure 5B**). Together, the data demonstrate that the immunostaining pattern seen in cells expressing of F805C-Kv11.1 protein is also seen in cells expressing other LQT2-linked mutant proteins in the CNBD.

### G601S and F805C Show Distinct Immunostaining Patterns in iPSC-CMs

**FIGURE 6:**
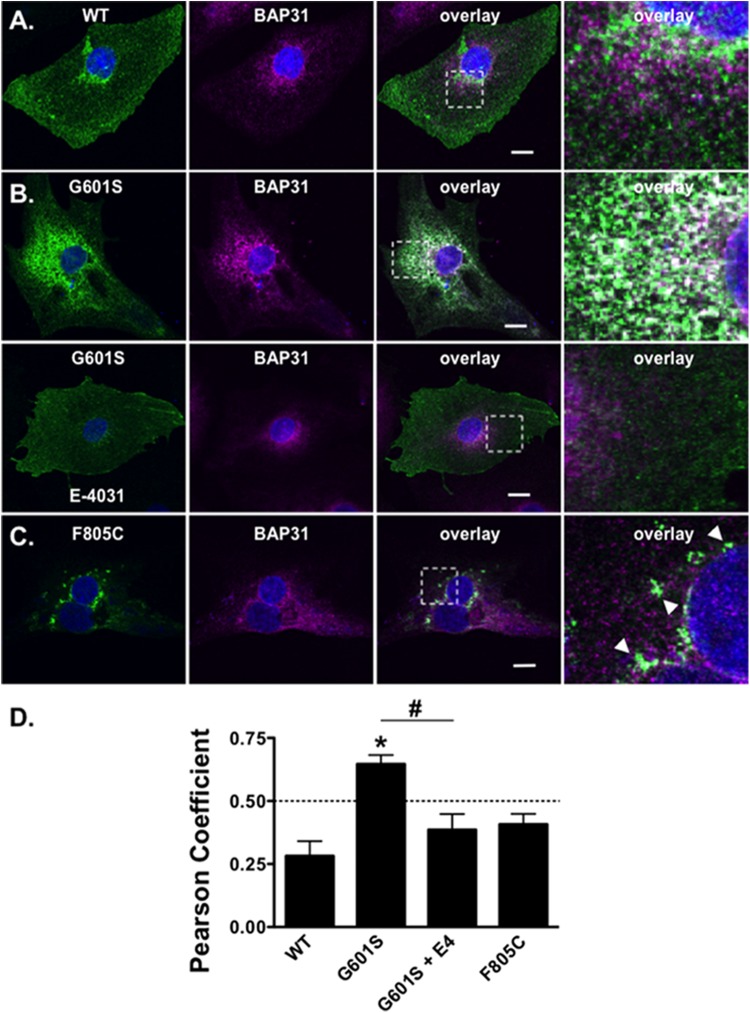
G601S- and F805C-Kv11.1 proteins show distinct immunostaining patterns in iPSC-CMs. Shown are representative images of iPSC-CMs expressing **(A)** WT-Kv11.1-GFP, **(B)** G601S-Kv11.1-GFP, or **(C)** F805C-Kv11.1-GFP (green, first column), immunostained with anti-BAP31 (purple, second column). Also shown are the overlays (co-localization is shown as white, third column) and the white dashed box portion of the overlay in more detail (fourth column). The nuclei are stained in blue and the scale bar represents 10 μm. The row in **(A)**, top row in **(B)**, and row in **(C)** show iPSC-CMs in control conditions. The arrowheads in **(C)** highlight the unique fluorescence pattern of cells expressing F805C-Kv11.1-GFP. The bottom row in **(B)** shows images from iPSC-CMs expressing G601S-Kv11.1-GFP after incubation in 10 μM E-4031 (E4) for ∼24 h. **(D)** The mean Costes’ Pearson Coefficient (PC) for the iPSC-CMs expressing WT-GFP (*n* = 16 images), G601S-GFP (*n* = 20 images), G601S-GFP after incubation in 10 μM E-4031 (*n* = 19 images), or F805C-GFP (*n* = 14 images) (^∗^*p* < 0.05 compared to WT, ^#^*p* < 0.05 compared to G601S con). Two independent cultures were tested for each condition.

We next determined whether the mutation-specific immunostaining patterns of G601S- or F805C-Kv11.1 proteins persisted in human inducible pluripotent stem cell derived cardiomyocytes (iPSC-CMs). This will help to determine if mutation-specific differences occurs in a more native-like cell that expresses endogenous WT-Kv11.1 protein (including the shorter Kv11.1b subunit) (Jones et al., 2004, 2014). iPSC-CMs were transfected with WT-, G601S- or F805C-Kv11.1 proteins tagged with GFP on the Kv11.1 amino-terminus to distinguish between heterologously expressed and endogenous WT-Kv11.1 or WT-Kv11.1b protein. We immunostained the cells for BAP31. Similar to what we previously showed in HEK293 cells, WT-Kv11.1-GFP protein expressed in iPSC-CMs did not co-localize with BAP31 (**Figures 6A,D**) (Smith et al., 2013). G601S-Kv11.1-GFP expressed in iPSC-CMs co-localized with BAP31, and importantly, this co-localization was decreased in iPSC-CMs treated with the pharmacological chaperone E-4031 for 24–48 h (**Figures 6B,D**). In most iPSC-CMs, F805C-Kv11.1-GFP protein concentrated in discrete compartments (although the co-localization with BAP31 was not as strong as it was in HEK293 cells) (**Figures 6C,D**). These data confirm that the differences in subcellular localization of WT-, G601S-, and F805C-Kv11.1 proteins persisted in a cell model of human cardiomyocytes.

## Discussion

In this study we demonstrate that the trafficking-deficient LQT2 missense mutation F805C generated Kv11.1 protein that accumulates in discrete ER subcompartments. In HEK293 cells F805C-Kv11.1 protein did not co-localize with several ER/ERGIC marker proteins, but it did co-localize with the transitional ER protein BAP31. The immunostaining pattern of F805C-Kv11.1 protein was sensitive to the microtubule depolymerizing drug nocodazole, as well as the proteasome inhibitors MG132 or lactacystin. Interestingly, these drugs did not alter F805C-Kv11.1 protein co-localization with BAP31, but they all caused the F805C-Kv11.1 protein to become more diffuse and increased the overlapping immunostaining between F805C-Kv11.1 and BAP31. In contrast, G601S-Kv11.1 protein did not accumulate in distinct subcompartments and its immunostaining pattern was insensitive to proteasome inhibition. Importantly, the mutation-specific differences in the subcellular patterns of G601S- and Kv11.1-Kv11.1 protein were also present in human iPSC-CMs. We conclude that distinct QC mechanisms in the ER regulate the retention of G601S- and F805C-Kv11.1 protein.

Mutation-specific differences in trafficking-deficient phenotype for LQT2 mutations have long been recognized (Ficker et al., 2000b; Anderson et al., 2006, 2014; Smith et al., 2016). For example, there are mutation-specific differences in pharmacological correction, intragenic suppression, and dominant negative effects on the trafficking of WT-Kv11.1 protein, suggesting that different mutations likely disrupt different steps in the native Kv11.1 protein folding pathway (e.g., co-assembly of α-subunits, formation of the voltage-sensor/pore domains, increasing the proximity of the NH_2_ and COOH termini, etc.) (Ficker et al., 2000a; Delisle et al., 2005; Anderson et al., 2006; Smith et al., 2016). However, this is the first study to investigate mutation specific differences in cellular mechanisms associated with ER retention. Although we focus only on two different mutations, we found that other trafficking-deficient LQT2-linked proteins have immunostaining/co-localization patterns similar to cells expressing G601S-Kv11.1 (A614V and N629D) (Smith et al., 2013) or F805C-Kv11.1 protein (R752W, **Figure 5B**). Therefore, this study has identified a novel way to categorize subgroups of trafficking-deficient LQT2 mutations. We expect these findings will help identify new drugs that improve LQT2 protein trafficking by targeting mutation-specific ER QC mechanisms.

The exact mechanism by which proteasome inhibitors alter F805C-Kv11.1 protein immunostaining remains unclear. Previous studies show that retrotranslocation of misfolded proteins into the cytosol likely occurs concurrently with their degradation by the proteasome, and that upon inhibition of proteasomal activity, misfolded proteins remain intact in the secretory pathway (Hirsch and Ploegh, 2000). Consistent with this concept, studies show that inhibition of the proteasome decreases the efficiency of WT-Kv11.1 protein dislocation from the ER (Gong et al., 2005). However, F805C-Kv11.1 protein did not co-localize with the ERAD protein derlin-1, and surprisingly, we found that longer-term proteasome inhibition did not improve Kv11.1 protein trafficking but actually appeared to inhibit it (data not shown).

An intriguing implication to the findings in this study is that, because different immunostaining patterns of different LQT2-linked mutant proteins can be easily visualized with microtubule depolymerizing agents or proteasome inhibitors, cell lines expressing these trafficking-deficient Kv11.1 proteins might be used as a novel drug-screening platform to identify new anti-neoplastic agents that have microtubule depolymerization properties or inhibit the proteasome activity.

There are several limitations to this study. We primarily used HEK293 cells for most of the experiments in the study, however, we confirmed similar mutation-specific differences in the subcellular localization of G601S- and F805C-Kv11.1 proteins in iPSC-CMs. Interestingly the co-localization of F805C-Kv11.1 and BAP31 protein was decreased in iPSC-CM. We suspect that this difference might be due to cell type specific differences. For example, the subcellular localization of mutant F805C-Kv11.1 protein may be altered by endogenous WT-Kv11.1 proteins (including the Kv11.1b protein). However, F805C-Kv11.1 is not expected to co-assemble with other Kv11.1 subunits (Ficker et al., 2002). Another possibility is that the larger size of iPSC-CMs might help to better resolve the spatial differences between F805C-Kv11.1 protein and BAP31. Regardless, the data clearly show that both G601S- and F805C-Kv11.1 proteins have unique localization patterns in both cell types, suggesting they are regulated by different QC pathway interactions.

## Author Contributions

AH, JS, and BD performed the experiments and data analysis. CA, CE, TM, CJ, and BD contributed to the writing, interpretation, editing, and presentation of the manuscript.

## Conflict of Interest Statement

The authors declare that the research was conducted in the absence of any commercial or financial relationships that could be construed as a potential conflict of interest.

## Footnotes

1 https://imagej.nih.gov
